# The incidence, mortality and disease burden of cardiovascular diseases in China: a comparative study with the United States and Japan based on the GBD 2019 time trend analysis

**DOI:** 10.3389/fcvm.2024.1408487

**Published:** 2024-09-18

**Authors:** Menglan Zhu, Wenyu Jin, Wangbiao He, Lulu Zhang

**Affiliations:** ^1^ Sanitation Teaching and Research Section of Department of Health Service, Naval Medical University, Shanghai, China; ^2^ Otolaryngology Department of Unit 32265 of the People’s Liberation Army, Guangzhou, China; ^3^ Cardiorenal Department of 79th Army Hospital, Liaoyang, China

**Keywords:** CVDs, ASIR, ASDR, disability-adjusted life years, GBD 2019, China, the United States, Japan

## Abstract

**Background:**

Cardiovascular diseases (CVDs) are not only the primary cause of mortality in China but also represent a significant financial burden. The World Health Organization highlight that as China undergoes rapid socioeconomic development, its disease spectrum is gradually shifting towards that of developed countries, with increasing prevalence of lifestyle-related diseases such as ischemic heart disease and stroke. We reviewed the rates and trends of CVDs incidence, mortality and disability-adjusted life years (DALYs) burden in China and compared them with those in the United States (US) and Japan for formulating CVDs control policies.

**Methods:**

Data on CVDs incidence, death and DALYs in China, the US and Japan were obtained from the GBD 2019 database. The Joinpoint regression model was used to analyze the trends in CVDs incidence and mortality in China, the US and Japan, calculate the annual percentage change and determine the best-fitting inflection points.

**Results:**

In 2019, there were approximately 12,341,074 new diagnosed cases of CVDs in China, with 4,584,273 CVDs related deaths, causing 91,933,122 DALYs. The CVDs age-standardized incidence rate (ASIR) in China (538.10/100,000) was lower than that in the US and globally, while age-standardized death rate (ASDR) (276.9/100,000) and age-standardized DALY rate (6,463.47/100,000) were higher than those in the two regions. Compared with the US and Japan, from 1990 to 2019, the CVDs incidence rate in China showed an increasing trend, with a lower annual decrease in ASDR and a younger age structure of disease burden. Furthermore, the disease spectrum in China changed minimally, with stroke, ischemic heart disease, and hypertensive heart disease being the top three leading CVDs diseases in terms of incidence and disease burden, also being the major causes of CVDs in the US and Japan.

**Conclusion:**

The prevention and control of CVDs is a global issue. The aging population and increasing unhealthy lifestyles will continue to increase the burden in China. Therefore, relevant departments in China should reference the established practices for CVDs control in developed countries while considering the diversity of CVDs in different regions when adjusting national CVDs control programs.

## Introduction

Cardiovascular diseases (CVDs) continues to be the predominant contributor to the global disease burden (1). The incidence, mortality, and disability-adjusted life years (DALYs) of CVDs vary greatly between countries and regions, especially between developing and developed countries (2). The burden of CVDs varies across different ages, genders, and countries/regions globally (3).

In nearly all countries outside of high-income countries, the burden of CVDs has been steadily increasing for decades. Surprisingly, in some areas where the age-standardized incidence of CVDs had previously decreased, it has now started to rise. Urgent emphasis on implementing existing cost-effective policies and interventions is essential to achieve the specific targets under Sustainable Development Goal 3 and reduce premature mortality from non-communicable diseases by 30%.Various variables, including ecological, environmental, demographic, cultural, and genetic factors, contribute to the heterogeneity of CVDs incidence, mortality, and DALY burden in different regions (4).

As the largest developing country in the world, China has a huge burden of CVDs (5). The epidemiology of CVDs in China is undergoing major changes: the burden of atherosclerotic CVDs is rising, the mortality of hemorrhagic stroke is decreasing, the regional prevalence trend of CVDs is different, the number of patients with moderate Ischemic Heart Disease (IHD) and stroke is increasing and the aging of cardiovascular patients is increasing (6). In order to adapt to China's current social and economic conditions and the development of an aging population, and successfully achieve the goal of “Healthy China 2030”, China must establish and improve an effective CVDs control system to develop a new model to prevent and control CVDs and related health problems.

Previously, in 2010, the American Heart Association announced a strategic goal to reduce total CVDs mortality by 20% within 10 years, selecting seven important CVDs modifiers and dubbed “Life's Simple” (7). Subsequently, the mortality of coronary heart disease and stroke in the US decreased significantly (8). As a super-aging society, Japan ranks the highest in the world in terms of population longevity, which is attributed to the effective control of CVDs, especially the significant reduction of stroke mortality (9, 10).

The prevention and control strategies of CVDs in the US and Japan have positive reference significance for China. Therefore, comparing the incidence, mortality and DALYs of CVDs between China and these developed countries, and establishing our cardiovascular health indicators based on the identified variable factors can provide effective guidance for CVDs prevention and adjustment control strategies in China.

## Materials and methods

### Data sources

This study utilized the Global Health Data Exchange (GHDx) online database (http://ghdx.healthdata.org) and employed the Global Health Data Exchange query tool (http://ghdx.healthdata.org/gbd-results-tool) to extract the incidence, death, and DALYs of CVDs for 21 age groups by sex (male and female) in China, the United States, and Japan from 1990 to 2019.

The GBD 2019 online database encompass 285 causes of death across 195 countries and territories from 1980 to 2019. The age-standardized incidence rate and age-standardized death rate (ASIR and ASDR) are computed using the world standard population as a reference (11). The DALYs attributable to CVDs in China, the US, and Japan in 1990 and 2019 were accessed through the GBD 2019 online results tool developed by the Institute for Health Metrics and Evaluation (IHME). Age standardized DALY rates are determined using the GBD reference population (12). The changes in percentage and ranking of all-age and age-standardized DALY rates from 1990 to 2019 depict the trends in the burden of CVDs in China, the US and Japan.

### Observational indicators

The incidence, mortality, and corresponding age-standardized rates of CVDs were quantified using publicly available data from the GBD 2019 database. DALYs were used to quantify the disease burden. Where the incidence rate = the number of new cases of a disease in a certain population during a certain period/the population at risk during the same period × K. The mortality rate = the total number of deaths in a certain population in a given year/the average population for that year in the same populatio*n* × K (13). DALY refers to the total years of healthy life lost from the onset of a disease to death, which is the sum of years lost due to disability (YLD) and years of life lost (YLL). YLD = number of cases × disease weight × average years lived with disability. YLL = standard life expectancy—age at death (14).

### Statistical analysis

We used Excel 2019 for statistical analysis and data processing. I analyzed the current situation and changes in the burden of CVDs using the change rate from 1990 to 2019. The change rate calculation formula is (2019 data—1990 data)/1990 data × 100. The Joinpoint regression model (15) was used to study the time trends of ASIR in China, the US and Japan from 1990 to 2019. Joinpoint software (version 5.0.2) was used to understand the time trends in a structured manner and test the statistically significant trends between the joining points. The model applied a maximum of three line segments (two joining points). In order to elucidate the direction and extent of the trends, this investigation computed the APC (16). In cases where the APC significantly deviates from zero, the terms “decrease” or “increase” are employed. A significance level of *P* < 0.05 is deemed statistically significant (17).

The Joinpoint model uses the Poisson variation model to estimate the trend data of the incidence rate. Significance testing is done using the Monte Carlo permutation method (18). In this study, we calculated the APC, average annual percentage change rate (AAPC), which calculated with geometric mean weighted APC, and the corresponding 95% confidence interval (CI) of the ASIR of CVDs in China, the US and Japan from 1990 to 2015 using Joinpoint Regression Program Version 4.5.0 (19).

### Ethical statement

This study is conducted in accordance with the guidelines of the Helsinki Declaration of 1975 and is exempt from ethical review and informed consent because of the use of anonymous public data sets.

## Results

### CVDs incidence and mortality in China, the US and Japan

In 2019, the global burden of CVDs was significant, with 55,451,741 reported cases worldwide. Of these, 22.26% occurred in China (men 45.94% and women 54.06%). The ASIR for CVDs in China is 652.21/100,000, with a slightly lower rate in male (645.30/100,000) compared to female (667.28/100,000).

In the same year, the US reported 3,693,571 new cases (men 50.88% and women 49.12%), while Japan reported 1,537,275 cases (men 42.79% and women 57.21%). The CVDs incidence in China was higher than in Japan (538.10/100,000), but lower than the global average (684.33/100,000) and the US (693.48/100,000). The top three CVDs types in new cases in China were stroke (200.84/100,000), ischemic heart disease (197.39/100,000), and peripheral artery disease (125.43/100,000) Supplementary Table S1.

**Figure 1:**
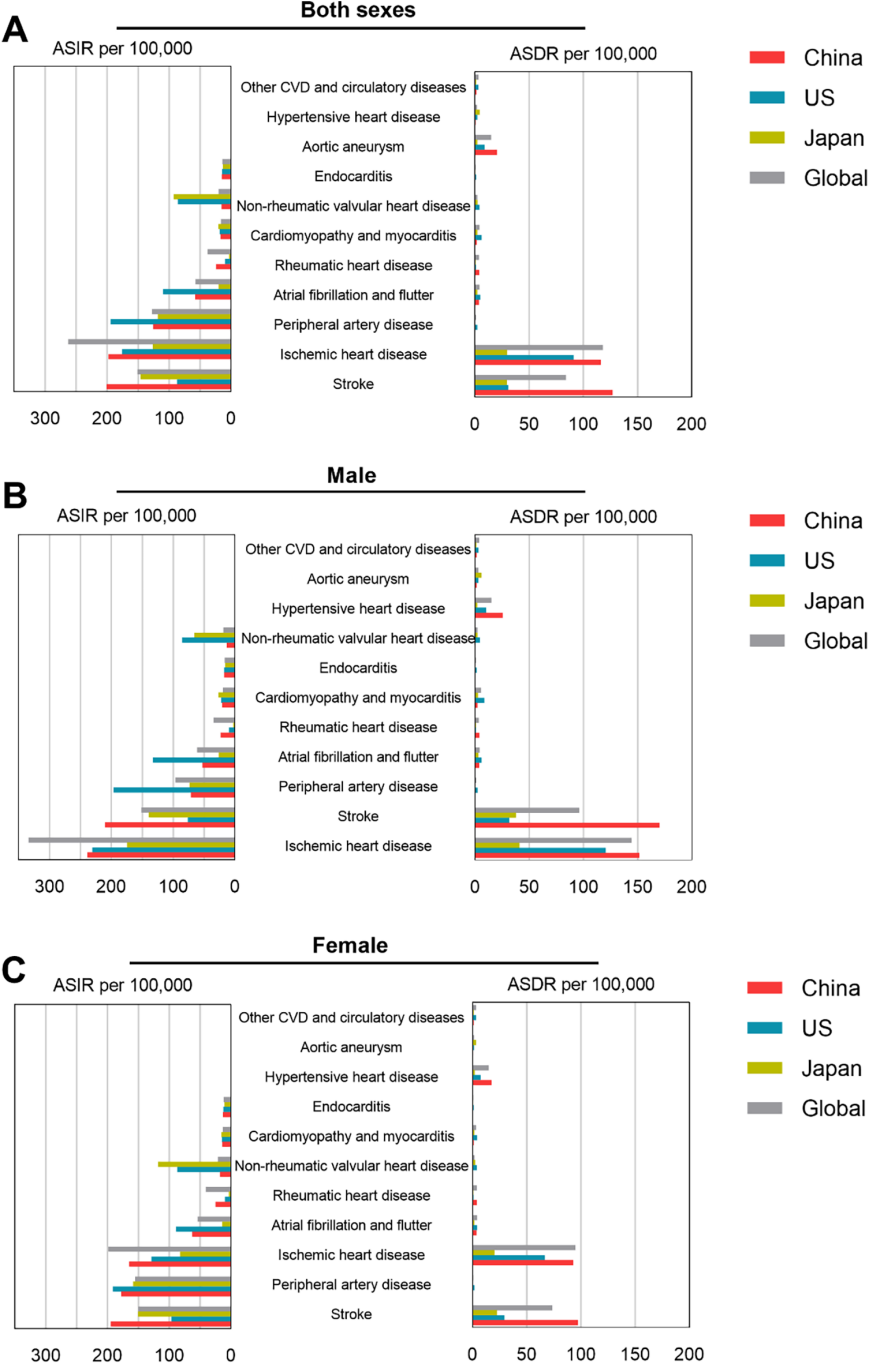
ASIR and ASDR per 100,000 population of selected types of CVDs in China, US, Japan and global in 2019. ASIR and ASDR data of CVDs in both sexes **(A)**, male **(B)**, and female **(C)**.

Notably, the stroke incidence in the US (86.96/100,000) was significantly lower than in China (200.84/100,000), Japan (145.78/100,000), and global (150.77/100,000) Figure 1A.

In 2019, the global number of deaths from CVDs was 18,562,510 (9,625,979 men, 8,936,530 women), with approximately 4,584,273 deaths in China (2,543,155 men, 2,041,118 women), accounting for 25.7% of all CVDs deaths worldwide. The number of deaths in the US was 957,455 (482,827 men, 474,628 women), and in Japan, it was 372,483 (169,703 men, 202,780 women) Supplementary Table S2.

The 2019 ASDR for CVDs in China, the US, and Japan was 276.90/100,000, 157.02/100,000, and 77.02/100,000 respectively. The ASDR for males is 361.90/100,000, and for females it is 219.70/100,000. China's ASDR is much higher than that of the US and Japan, slightly higher than the global average. Stroke, ischemic heart disease, and hypertensive heart disease are the three most important CVDs in China, with ASDR in 2019 of 127.2/100,000, 116.4/100,000, and 20.58/100,000, as shown in Figure 1A; Supplementary Table S2.

### Time trends of CVDs incidence and mortality in China, the US, and Japan, 1990–2019

**Figure 2:**
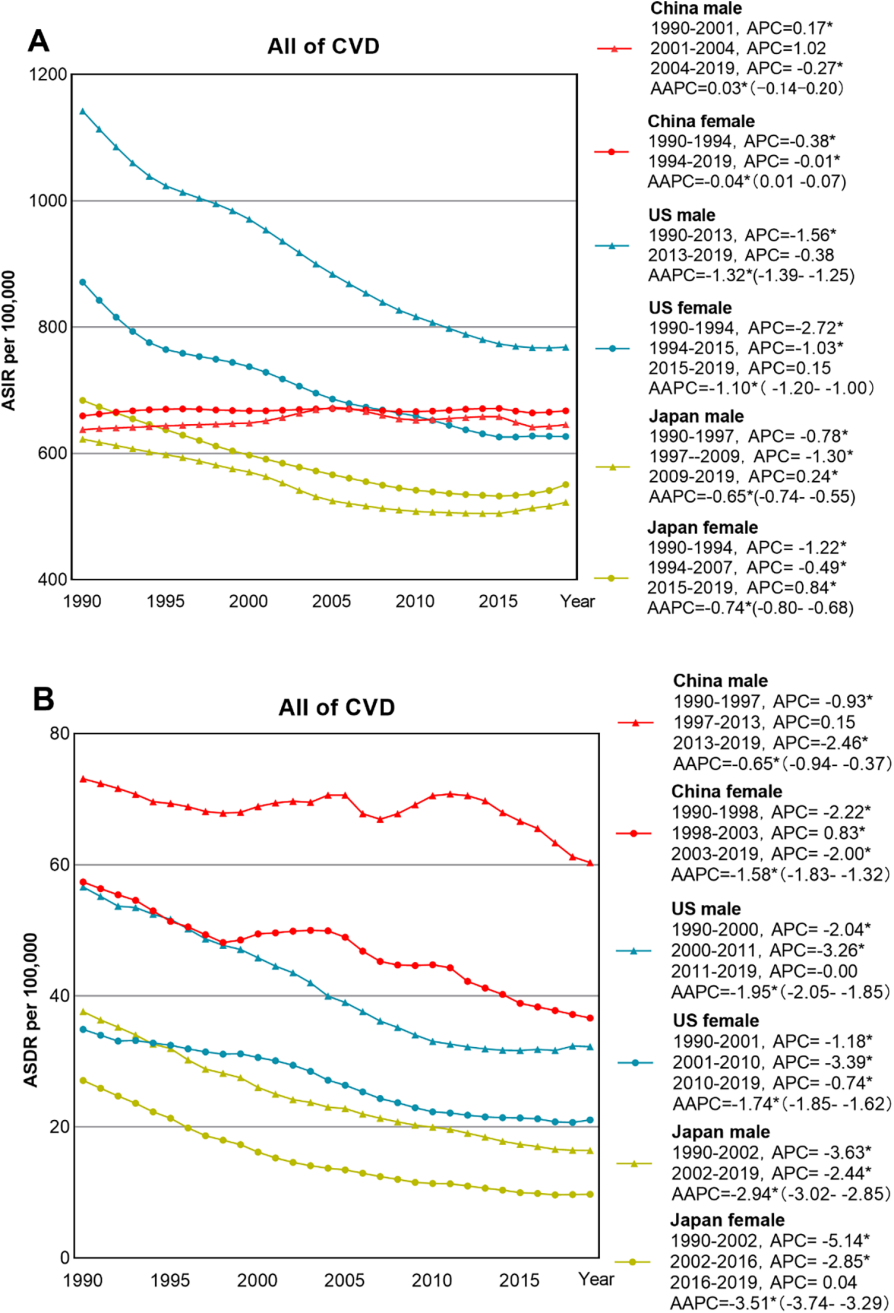
Trends in ASIR **(A)** and ASDR **(B)** of all of CVDs sites by gender in China, US and Japan.

The ASIR of CVDs in China showed an overall fluctuating upward trend, increasing by 0.03% (95% CI: −0.08 to 0.13%) per year from 1990 to 2019. However, the ASIR of CVDs in the US and Japan decreased significantly by −1.23% (95% CI: −1.3 to −1.10%) per year and −0.70% (95% CI: −0.74 to −0.67%) per year. The ASIR of CVDs in China increased from 646.20/100,000 to 652.21/100,000 (an increase of 0.93%) from 1990 to 2019, with the ASIR for males rising from 637.78/100,000 to 645.30/100,000 (an increase of 1.18%) from 1990 to 2019, and the ASIR for females increased from 659.22/100,000 to 667.28/100,000 (an increase of 1.22%) from 1990 to 2019, Analysis of Variance did not reveal any statistical significance (*P *> 0.05) (Figure 2).

The ASDR for CVDs in China, the US, and Japan decreased annually by 1.01% (95% CI: −1.33 to −0.69%), −1.82% (95% CI: −1.9 to −1.72%), and −3.07% (95% CI: −3.30 to 2.84%) respectively from 1990 to 2019. Japan showed the most significant decline. The ASDR for all CVDs in Chinese males decreased from 438.64/100,000 to 361.93/100,000, and the ASDR for Chinese females decreased from 344.30/100,000 to 219.73/100,000 with no statistical significance by Analysis of Variance (*P > 0.05*), Table 1.

**Table 1:** The temporal trend of the incidence and death rate of CVDs in China, Japan, the USA, and the world from 1990 to 2019.

	China		US		Japan		Global	
	AAPC (%)	95%CI (%)	AAPC (%)	96%CI (%)	AAPC (%)	97%CI (%)	AAPC (%)	98%CI (%)
ASIR								
Both	0.03	(−0.08 to 0.13)	−1.23*	(−1.35 to 1.10)	−0.70*	(−0.74 to 0.67)	−0.52*	(−0.59 to 0.45)
Male	0.03	(−0.14 to 0.20)	−1.32*	(−1.39 to 1.25)	−0.65*	(−0.74 to 0.55)	−0.51*	(−0.59 to 0.43)
Female	0.04*	(0.01 to 0.07)	−1.10*	(−1.20 to 1.00)	−0.74*	(−0.80 to 0.68)	−0.51*	(−0.57 to 0.46)
ASDR								
Both	−1.01*	(−1.33 to 0.69)	−1.82*	(−1.92 to 1.72)	−3.07*	(−3.30 to 2.84)	−1.38*	(−1.48 to 1.29)
Male	−0.65*	(−0.94 to 0.37)	−1.95*	(−2.05 to 1.85)	−2.94*	(−3.02 to 2.85)	−1.27*	(−1.40 to 1.15)
Female	−1.58*	(−1.83 to 1.32)	−1.74*	(−1.85 to 1.62)	−3.51*	(−3.74 to 3.29)	−1.52*	(−1.62 to 1.43)

* Statistically significant (*p* < 0.05); AAPC, average annual percent change.

**Figure 3:**
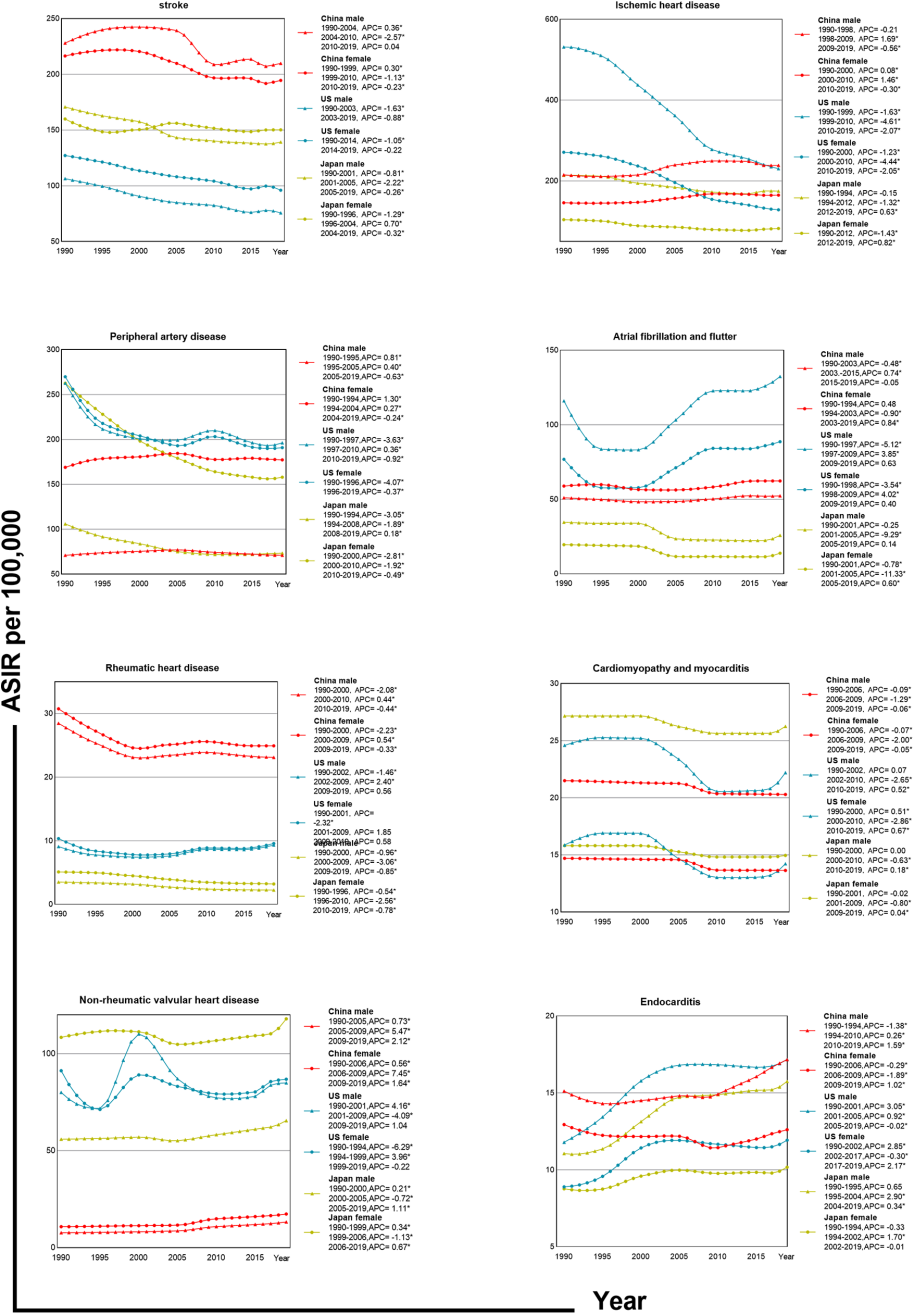
Trends in ASIR by CVDs site and gender in China, US and Japan.

Joinpoint analysis shows that ASIR changes in Chinese males had three stages; the most rapid growth was during 2001–2004 (APC = 1.02), while females had their peak growth from 1990 to 1994 (APC = 0.38). In contrast, American males saw the sharpest decline from 1990 to 2013 (APC = −1.56), and for females, it was from 1990 to 1994 (APC = −2.72). Japanese males experienced the fastest decline from 1997 to 2009 (APC = −1.30), and Japanese females from 1990 to 1994 (APC = −1.22). The ASDR changes for Chinese males and females were also segmented into three phases with males declining the fastest from 2013 to 2019 (APC = −2.46), and females from 1990 to 1998 (APC = −2.22). The curves of CVD subcategory incidence rates in China, the US, and Japan exhibit notable discrepancies (Figure 3).

### China’s CVDs burden and comparison with the US and Japan

**Figure 4:**
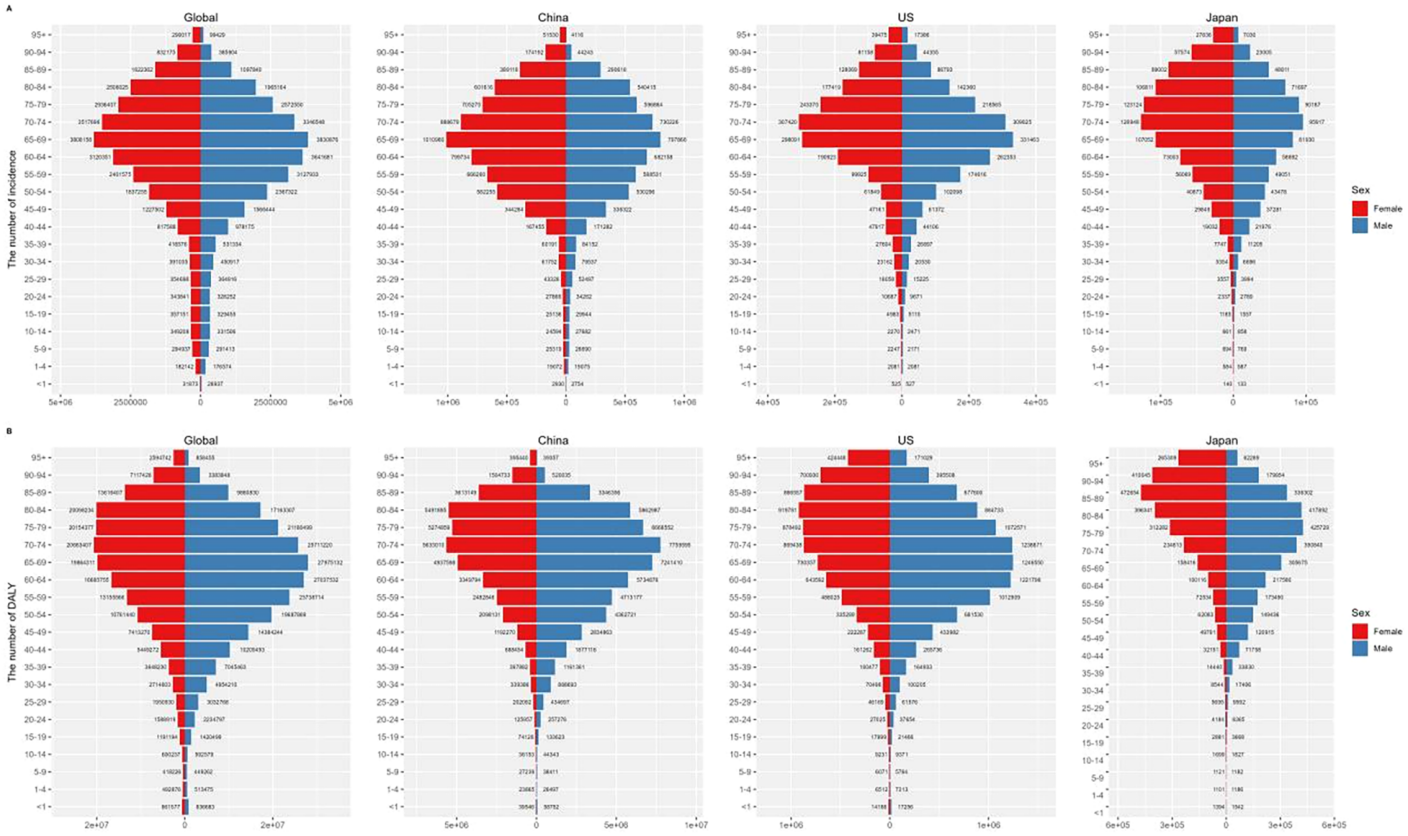
Numbers of incidence **(A)** and DALYs **(B)** of CVDs by age groups in worldwide, China, the US, and Japan in 2019.

**Figure 5:**
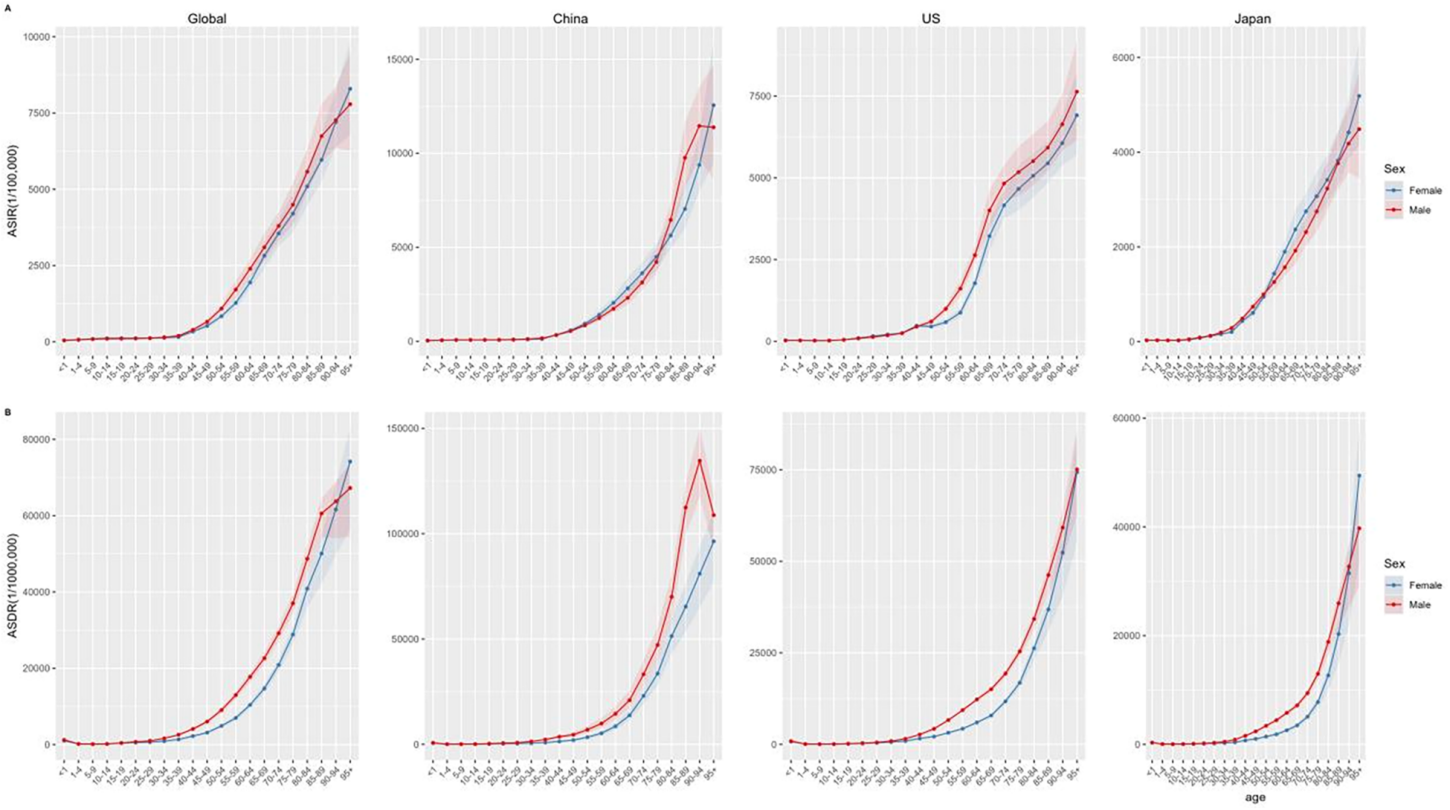
ASIR **(A)** and ASDR **(B)** of CVDs by age groups in worldwide, China, the US, and Japan in 2019.

In 2019, the global burden of disease was estimated to be 393,107,482 DALYs, with China accounting for 91,933,122 DALYs, the US for 17,266,977 DALYs and Japan for 5,536,050 DALYs (Figure 4). For all CVDs, China's age-standardized DALY rate was 6,463.47/100,000, while the US and Japan had rates of 5,264.66 and 4,332.20/100,000, respectively (Supplementary Table S3). China's age-standardized DALY rate/100,000 was higher than that of the US and Japan (Figure 5).

**Figure 6:**
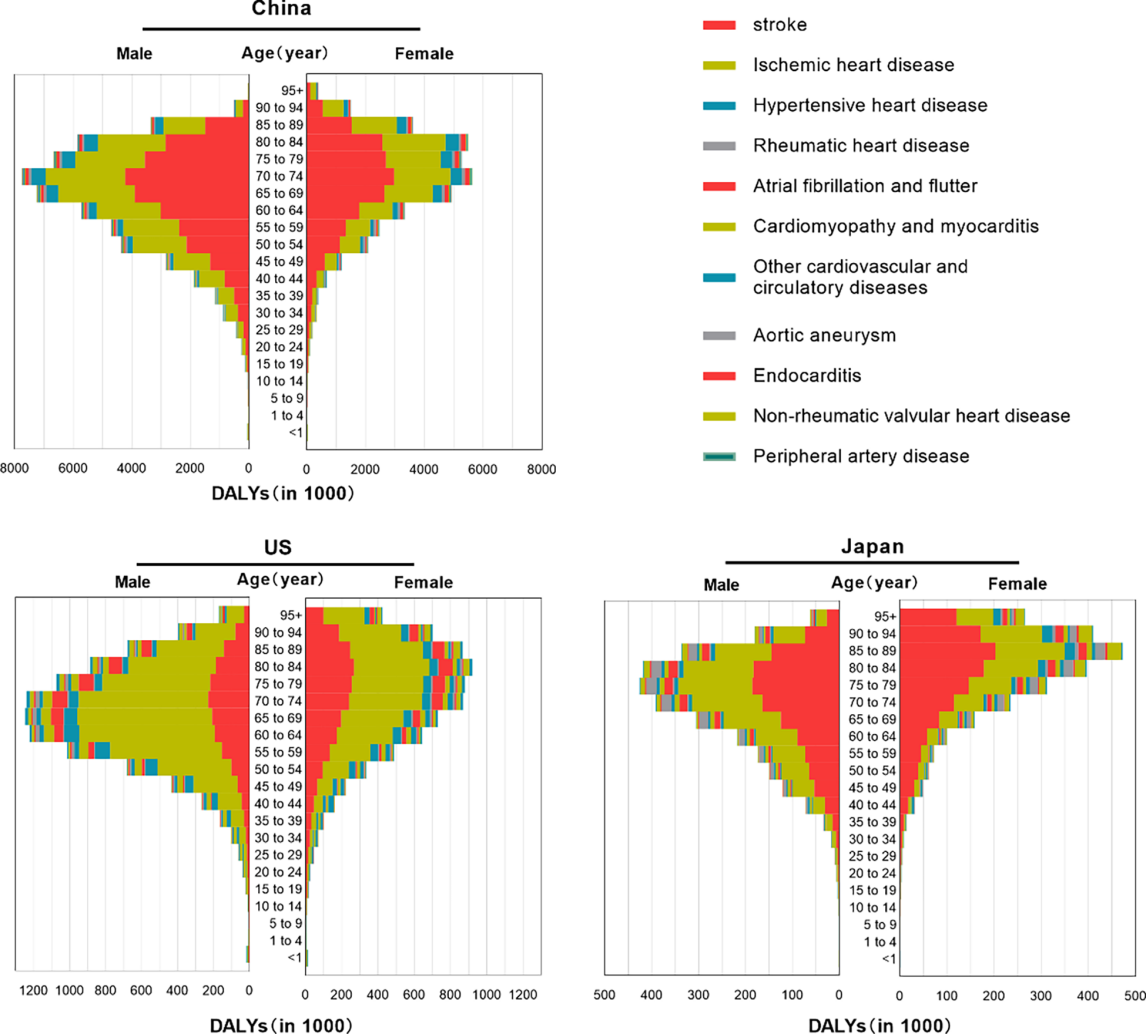
Numbers of DALYs attributable to CVDs by age and gender in China, US and Japan in 2019.

Stroke was the leading cause of DALYs in China (45,949,134 years, accounting for 49.98% of all CVDs DALYs), followed by ischemic heart disease (34,685,806 DALYs; 37.73%) and hypertensive heart disease (5,594,910 DALYs; 6.09%). The burden of disease for males in all age groups was higher than that for females (Figure 6).

Stroke and ischemic heart disease were the major contributors to the CVDs burden in these three countries (Supplementary Table S3).

### Changes in CVDs burden in China, the US and Japan

The age-standardized DALY rate in China decreased by 33.4% during this period, with notable decreases in ASDR for both males (24.6%) and females (42.6%). The ranking of CVDs types by DALY rate showed that stroke, ischemic heart disease, and hypertensive heart disease maintained their positions as the leading causes, with varying degrees of decline in DALY rates. Additionally, atrial fibrillation and flutter, cardiomyopathy and myocarditis, non-rheumatic valvular heart disease, and peripheral artery disease showed an increase in ranking, while rheumatic heart disease, other cardiovascular and circulatory diseases, and endocarditis experienced a decrease in all-age DALY rates over the 29-year period.

**Figure 7:**
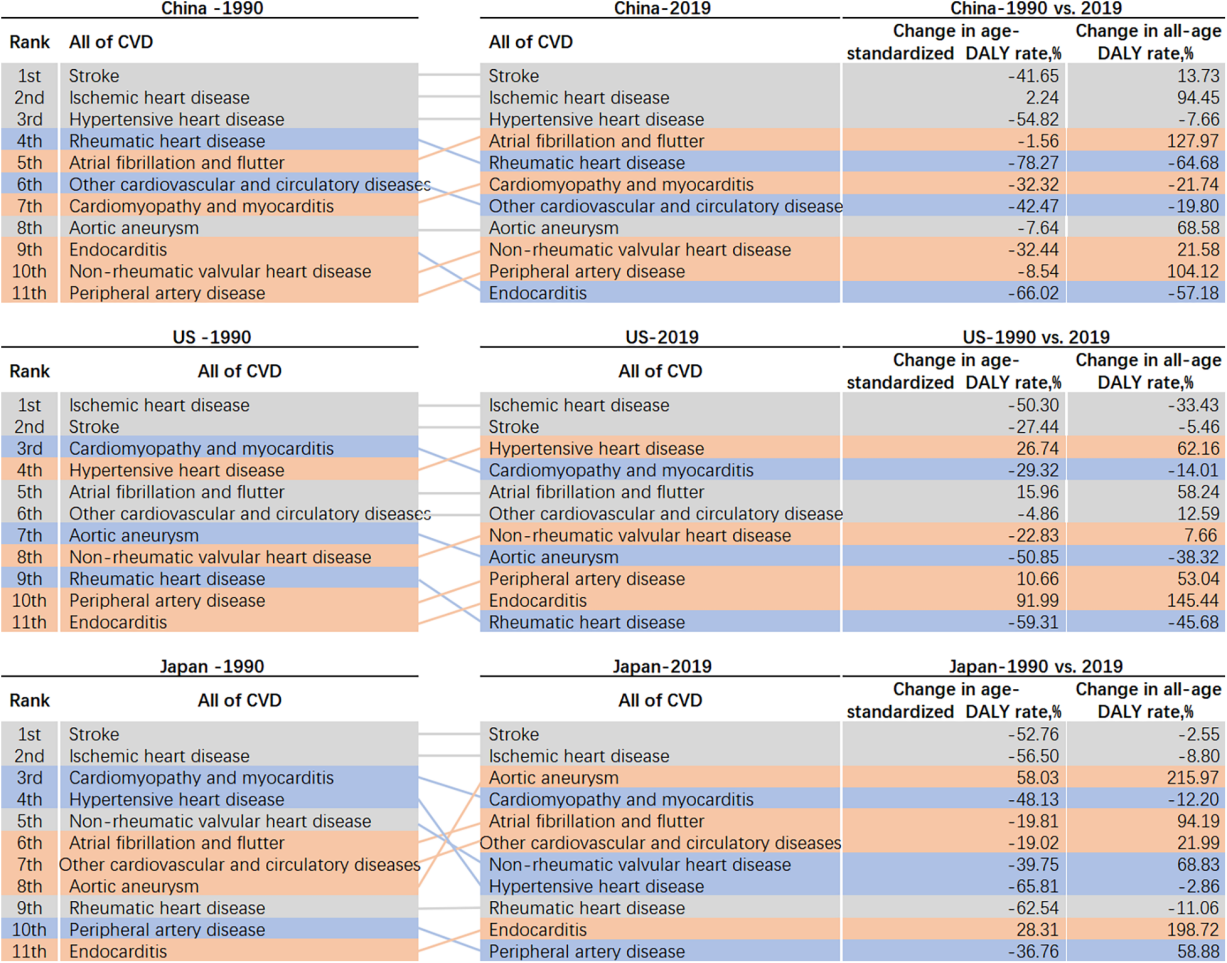
Rank changes in DALY attributable to CVDs and percentage change in all age and age-standardized DALY rates.

Similar to the burden of CVDs in China, from 1990 to 2019, ischemic heart disease, stroke, and hypertensive heart disease consistently ranked as the top three causes in the US, with DALY rates varying across age groups. During the same period in Japan, stroke and ischemic heart disease remained the first and second leading causes of death, while hypertensive heart disease dropped from fourth to seventh place in 2019, with a 65.81% reduction in DALY rates across all age groups (Figure 7).

## Discussion

### Epidemiological analysis and trends of CVDs in China

Based on the latest data from GBD 2019, we conducted a comprehensive epidemiological analysis of the incidence, mortality, and DALY of CVDs in China, focusing on the temporal trends of CVDs incidence, mortality, and DALY burden from 1990 to 2019, and comparing with the US and Japan. The study results reveal that although the age-standardized incidence of CVDs in China is lower than that of the US and Japan, the ASDR and DALY rates of CVDs are higher.The change in burden of CVDs in China, with comparison to the US and Japan, from 1990 to 2019 has been significant.

The incidence of CVDs in China has been increasing year by year, with a slower decline in mortality rate, and a younger age structure of disease burden (20). Major CVDs diseases in China include stroke, ischemic heart disease, and hypertensive heart disease, which are similar to the major causes in the US and globally. Additionally, the burden of atrial fibrillation and flutter, cardiomyopathy and myocarditis, non-rheumatic valvular heart disease, and peripheral arterial disease has also increased.

In China, the mortality rate of CVDs accounts for 46.74%–44.26% of all deaths in rural and urban areas, respectively (21). A significant feature of the CVDs epidemic in China is the rapid growth of atherosclerotic cardiovascular diseases (ASCVDs), which is related to the increased risk of coronary heart disease in middle-aged and older adult individuals, making them the focus of attention (22). Furthermore, the incidence of CVDs in Chinese women has surpassed that of men and continues to rise. Although the age-standardized incidence and mortality of CVDs in China have decreased since 1990, the disease burden of stroke remains very serious (23).

China has the highest stroke incidence rate, while the US has the lowest. Initially, the ASIR for American males surpassed China's for ischemic heart disease but later dropped significantly below the Chinese level. In the early stages of peripheral artery disease, both Chinese males and females experienced increasing ASIR until a decline commenced in 2004. ASIR for males and females in the US and Japan both trended downward.

### Global risk factors and health disparities related to CVDs

Gender and age are important influencing factors. The incidence rate of the same disease is generally higher in Chinese men than in women. Before menopause, women have a lower risk of CVD than men (24, 25). As age increases, the risk of CVD naturally rises due to vascular hardening and the accumulation of fatty deposits (26). The population bearing the highest burden of CVDs is younger in China compared to the US and Japan. Although traditionally considered a disease of the older adult, CVD is increasingly affecting younger populations due to lifestyle factors (27). Globally, the primary factors leading to loss of life from CVDs include dietary risks, tobacco exposure, elevated blood pressure, high body mass index, and increased fasting blood sugar levels (28, 29). Metabolic factors are the primary contributors to ischemic heart disease burden in the US (30, 31).

In terms of age composition, significant contributors to the CVD burden among children and adolescents in all three countries were myocardial and endocardial diseases, along with other cardiovascular and circulatory diseases.

Among the older adult in China, hypertensive heart disease was a major secondary contributor, while other cardiovascular and circulatory diseases were significant for young and older adult women in Japan. In Japan, the major secondary contributor for older adult men was aortic aneurysm, while in the US, myocardial and endocardial diseases greatly impacted young individuals, hypertensive heart disease affected middle-aged individuals, and atrial fibrillation and flutter were significant for the older adult.

Most individuals with acute myocardial infarction have at least one adverse cardiovascular risk factor prior to ASCVD (32). CVDs and related risk factors highlight global health disparities (33), with incidence and mortality differences across income levels and racial groups (1, 34).

Sociodemographic factors like race and location contribute to these disparities, and rural areas face greater challenges due to variations in healthcare and infrastructure (35). Addressing health determinants and ensuring resource accessibility are crucial for reducing CVD disparities (36).

### The comparison and experience sharing of CVDs prevention and management in the US, Japan, and China

In the US, the burden of CVDs is quite heavy, and through research and clinical intervention measures, the current stroke incidence rate in the US is much lower than in China (37). The US has implemented multiple measures in various areas to improve cardiovascular health, such as restricting the sale and use of tobacco products, requiring food producers to label nutritional information on packaging to reduce the intake of high cholesterol, high-fat foods (38), and promoting physical exercise and health policies to reduce the risk of CVDs. Effective prevention of CVDs can also be achieved through health education (39).

In some studies in the US, Le and his colleagues introduced a powerful interactive visualization tool that can determine county-level mortality rates and trends in several CVDs outcomes based on different sociodemographic characteristics (40). Implementing community-based approaches to reduce the risk of CVDs and move individuals towards ideal cardiovascular health is crucial for preventing many important health conditions (23, 41). For example, Long and his colleagues assessed a 3-year sodium reduction program in 3 community dietary programs in Arkansas (42). Smith and his colleagues studied the benefits of screening chronic health conditions (including blood pressure monitoring) using trusted community spaces (such as barber shops and beauty salons) in Arkansas (43).

Japan has implemented recent legislative measures targeting the resolution of challenges such as the deficiency in emergency stroke care personnel, public ignorance regarding stroke warning signs, and inadequate awareness regarding the necessity to summon an ambulance upon identification of stroke-like symptoms (44, 45). Emphasizing primary prevention to reduce disease morbidity, allocation of medical resources for CVDs emergencies and critical care, providing rehabilitation services, and secondary prevention to reduce the risk of recurrence, rehospitalization, and disability of CVDs survivors are essential (46).

China is undergoing an unhealthy shift in lifestyle, such as an increase in obesity, excessive consumption of red meat, and sedentary behavior, contributing to the increasing incidence of CVDs (47). The burden of stroke in China can be alleviated through blood pressure management, lifestyle interventions, and air pollution control (39). In addition, Asia lacks a large-scale stroke registration system (48), and recommends establishing a highly focused national CVDs center database to promote understanding of the actual situation of CVDs treatment in China and to reveal substantial differences in the burden and sub-burden of CVDs in various provinces in China (5). Primary and secondary CVDs prevention, especially in women (49), requires special attention. Formulating a national strategy focused on diagnosed women with CVDs to narrow the gap and obtain maximum benefits is particularly important.

### Limitations

The data for this study was obtained from the GBD database, which may have potential inaccuracies and biases that could affect the credibility of the study conclusions. Cross-national comparative studies may be influenced by different data collection and reporting standards in each country, making comparisons between data difficult and prone to errors. The factors contributing to these differences need further analysis to ensure that the conclusions have more guiding value.

## Conclusions

In our investigation, utilizing the most recent data from the GBD 2019, we performed a thorough epidemiological assessment of the incidence, mortality, and DALYs associated with CVDs in China. Furthermore, we examined the temporal patterns of CVDs incidence, mortality, and DALYs in China spanning the previous 29 years, and conducted comparisons with high-quality data sourced from the GBD database to delineate distinctions between China, the US and Japan.

Overall, the incidence of CVDs in China is slightly lower than in the US, but due to the large population in China, uneven regional development, and relatively lagging CVDs control strategies, the mortality and DALY rates of CVDs in China are much higher than in the US and Japan. Over the next decade, with the increasing aging population and unhealthy lifestyles, the burden of CVDs in China will continue to rise. Therefore, the Chinese government should fully promote cost-effective CVDs screening and immunization programs, implement strict tobacco control policies, and raise awareness of healthy lifestyles through education. At the same time, establishing a CVDs registry database is also crucial.
